# e14a2 Transcript Favors Treatment-Free Remission in Chronic Myeloid Leukemia When Associated with Longer Treatment with Tyrosine Kinase Inhibitors and Sustained Deep Molecular Response

**DOI:** 10.3390/jcm13030779

**Published:** 2024-01-29

**Authors:** Sílvia Marcé, Aleix Méndez, Blanca Xicoy, Natalia Estrada, Marta Cabezón, Antonella Luciana Sturla, Miriam Ratia García, Anna Angona, Paula Amat, Silvia Escribano Serrat, Emilia Scalzulli, Mireia Morgades, Alicia Senín, Juan Carlos Hernández-Boluda, Francisca Ferrer-Marín, Eduardo Anguita, Montserrat Cortés, Esther Plensa, Massimo Breccia, Valentín García-Gutierrez, Lurdes Zamora

**Affiliations:** 1 Hematology Department, Myeloid Neoplasms Group, ICO Badalona-Hospital Germans Trias i Pujol, Josep Carreras Leukaemia Research Institute (IJC), 08916 Badalona, Spain; emendezlopez@iconcologia.org (A.M.); bxicoy@iconcologia.net (B.X.); nestrada@carrerasresearch.org (N.E.); mcabezon@iconcologia.net (M.C.); mmorgades@iconcologia.net (M.M.); lzamora@iconcologia.net (L.Z.); 2 Hematology Department, ICO Hospitalet-Hospital Duran y Reynals, 08908 Barcelona, Spain; asturla@idibell.cat (A.L.S.); mratia@idibell.cat (M.R.G.); maseninm@iconcologia.net (A.S.); 3 Hematology Department, ICO Girona-Hospital Josep Trueta, 17007 Girona, Spain; angona.figueras@iconcologia.net; 4 Hematology Department, Hospital Clínico Universitario-INCLIVA de Valencia, 46010 Valencia, Spain; amat_pau@gva.es (P.A.); hernandez_jca@gva.es (J.C.H.-B.); 5 Hematology Department, Hospital Clínico San Carlos, IML, IdISSC, Universidad Complutense de Madrid (UCM), 28040 Madrid, Spain; sescribano@clinic.cat (S.E.S.); eduardo.anguita@salud.madrid.org (E.A.); 6 Hematology, Department of Precision and Translational Medicine, Policlinico Umberto 1, Sapienza University, 00189 Rome, Italy; scalzulli@bce.uniroma1.it (E.S.); breccia@bce.uniroma1.it (M.B.); 7 Hematology Department, Hospital General Universitario Morales Meseguer-CIBERER, IMIB, UCAM, 30008 Múrcia, Spain; fferrer@ucam.edu; 8 Hematology Department, Hospital General de Granollers, 08402 Granollers, Spain; mcortes@althaia.cat; 9 Hematology Department, Consorci Sanitari del Maresme, Hospital de Mataró, 08301 Mataró, Spain; mplensa@csdm.cat; 10 Hematology Department, Hospital Ramón y Cajal, IRYCIS, Universidad de Alcalalá Madrid, 28801 Madrid, Spain; josevalentin.garcia@salud.madrid.org

**Keywords:** chronic myeloid leukemia, *BCR::ABL1* transcript type, tyrosine kinase inhibitors, discontinuation, treatment free remission

## Abstract

e13a2 and e14a2 are the most frequent transcript types of the *BCR::ABL1* fusion gene in chronic myeloid leukemia (CML). The current goal with tyrosine kinase inhibitors (TKI) is to achieve sustained deep molecular response (DMR) in order to discontinue TKI treatment and remain in the so-called treatment-free remission (TFR) phase, but biological factors associated with these goals are not well established. This study aimed to determine the effect of transcript type on TFR in patients receiving frontline treatment with imatinib (IM) or second-generation TKI (2G-TKI). Patients treated at least 119 months with IM presented less post-discontinuation relapse than those that discontinued IM before 119 months (*p* = 0.005). In addition, cases with the e14a2 transcript type treated at least 119 months with IM presented a better TFR (*p* = 0.024). On the other hand, the type of transcript did not affect the cytogenetic or molecular response in 2G-TKI treated patients; however, the use of 2G-TKI may be associated with higher and earlier DMR in patients with the e14a2 transcript.

## 1. Introduction

Chronic myeloid leukemia (CML) is characterized by the presence of Philadelphia chromosome (Ph) containing the *BCR::ABL1* fusion gene with e13a2 and e14a2 as the most frequent transcript types. Imatinib (IM) became the standard tyrosine kinase inhibitor (TKI) of care for patients with chronic phase CML (CP-CML), demonstrating survival benefits and leading to a near-to-normal life expectancy. However, drug resistance or intolerance to IM in some patients led to the development of the second-generation (2G)TKIs dasatinib (DA), nilotinb (NIL) and bosutinib (BO), which presented a greater and faster efficacy [1]. Currently, IM, DA, NI and BO have been approved for frontline treatment of CP-CML [2], but lifelong treatment is associated with high cost and the adverse events related to these drugs affect the patients’ quality of life and treatment response [3,4]. In this sense, the current goal-standard in CML treatment is to achieve deep molecular response (DMR), MR^4.0^ or MR^4.5^, in order to discontinue TKI treatment and avoid the negative effects. Results from different trials suggest that patients with a sustained deep molecular response (sDMR) of at least 2 years can safely discontinue TKI [1,3,5,6,7].

Approximately 50% of patients with sDMR can remain relapse-free after discontinuing TKI treatment in the so-called treatment-free remission (TFR) phase, defined as the time from TKI discontinuation to the date of restarting therapy or the date of the last control if treatment is not restarted [8,9]. In this context, it is important to find factors at diagnosis that ensure a successful DMR, sDMR, and especially TFR. Some studies postulated that younger age and high Sokal [10] or EUTOS long-term survival (ELTS) [11] scores at diagnosis may contribute to the risk of relapse after discontinuation [6,12,13,14]. On the contrary, other studies demonstrated that the duration of treatment and the depth and duration of DMR before discontinuation were key factors for achieving TFR [7].

A TFR rate of 91% was calculated for patients who achieved MR^4.5^ or higher and remained on TKI treatment for more than 6 years [15]. There are few studies about the effect of the *BCR::ABL1* transcript type on TFR. Recently, some studies focused on the effect of transcript type in long-term TFR after TKI discontinuation and suggested a negative effect of the e13a2 transcript on a durable TFR and an association between longer TFR in CP-CML patients with e14a2 [16,17,18].

We recently published a trend to a longer TFR in CP-CML patients with e14a2 who discontinued IM in frontline treatment [8]. Evidence of the influence of transcript type on TFR in patients treated frontline with 2G-TKI is very scarce [1,6,12,13,18,19]. Most of them postulated a better response rates in those patients with the e14a2 transcript type.

The aim of the present study was to evaluate the effect of the e13a2 and e14a2 *BCR::ABL1* transcripts on DMR and TFR in a large series of CP-CML patients treated with IM and 2G-TKI.

## 2. Materials and Methods

### 2.1. Patient Samples

The study population consisted of 263 patients (aged >18 years) diagnosed with CP-CML from 1992 to 2021 who were treated with IM (224 patients), NIL (25 patients), DA (13 patients) or BO (1 patient) as frontline treatment. The present study adds 22 new patients treated with IM (10 with e13a2 and 12 with e14a2) to our previously published cohort [8]. Samples were collected in nine Spanish and one Italian center after informed consent accordingly with the Institutional Research Board protocols (Ref. CEI: PI-15-007, Code: ICO-ITK-2015-01 [ISOF-p210-LMC]) and the Declaration of Helsinki was signed.

At diagnosis and follow-up of IM, NIL or DA treatment, peripheral blood (PB) or bone marrow (BM) (when applicable) samples were analyzed. Patients records were used to gather demographic and clinical characteristics. Although patients were treated according to different European Leukemia Net (ELN) guidelines, over the study period, cytogenetic and molecular responses were analyzed according to the 2020 ELN guidelines [20]. During the TFR phase, patients were monitored monthly during the first 6 months, then every 6 weeks until the first year and every 3 months thereafter. Patients who had changed from IM to another TKI prior to DMR acquisition were not eligible for evaluation of the study endpoints.

### 2.2. Cytogenetic Studies

At diagnosis and at 3, 6 and 12 months (if applicable according to the guidelines prevailing at that time), conventional chromosome G-banding (CG-banding) was carried out for patients treated with IM and at 3 and 6 months for those receiving 2G-TKI.

Fluorescent “in situ” hybridization (FISH), according to the manufacturer’s instructions using LSI *BCR::ABL* dual-color, dual-fusion (Vysis-Abbott Molecular), was conducted at diagnosis when CG-banding was unprodcutive. A minimum of 200 interphase nuclei were analyzed from each case.

### 2.3. Determination of BCR::ABL1 Transcript Type and Real-Time Quantitative Polymerase Chain Reaction

EDTA tubes were used to collect PB or BM samples. *BCR::ABL1* was amplified using polymerase chain reaction (PCR) primers as previously described [21]. PCR products were transferred to a QIAxcel (QIAGEN Inc., Germantown, MD, USA) to identify the type of transcript expressed depending on its size.

Real-time quantitative PCR was carried out in an ABI7900 PCR thermal cycler (Applied Biosystems, Foster City, CA, USA). *BCR::ABL1* ratio was obtained as a percentage relative to an endogenous control gene expression (*ABL1*) according to the international scale (IS). The ratio was analyzed at diagnosis and during follow-up to determine the grade of molecular response and its duration. Molecular response was evaluated according to the ELN 2020 guidelines, when applicable. DMR was considered as the achievement of MR^4.0^ or deeper. Patients with sDMR for at least two years were eligible for TKI discontinuation.

### 2.4. Statistical Analysis

Frequency and percentage were used to describe baseline characteristics for categorical variables and median and range for quantitative variables. Chi-square or Fisher exact test, if applicable, were used to compare categorical variables between groups, while continuous variables were evaluated with the median test.

The cumulative incidence of molecular and cytogenetic response was defined as the achievement of such response with IM or 2G-TKI during the first 3, 6 and 12 months after treatment onset and was analyzed as frequency rates. The cumulative incidence of DMR was defined as the time from diagnosis until the time of MR^4.0^ or MR^4.5^ acquisition, which was considered as event, and was analyzed by competing risks, where the competitive events were patients who died or had changed TKI during the first 12 months without achieving response.

Logistic regression was used to analyzed the correlation between TKI discontinuation and transcript type. TFR was established as the time from IM or 2G-TKI discontinuation to the loss of major molecular response (MR^3.0^), restart of TKI treatment, progression, or death by any cause, considering the earliest of these events. The Kaplan–Meier method was performed to calculate TFR probabilities and the log-rank test was used to compare groups.

The R software (version 4.2.2) was used for all analyses, and two-sided *p* values < 0.05 were established as statistically significant.

## 3. Results

### 3.1. Effect of Transcript Type on DMR and TFR in IM-Treated Patients

Demographic and clinical data as well as cytogenetic and molecular responses according to the transcript type of patients treated with IM were reported previously [8]. Data of the whole IM cohort with 22 additional patients demonstrated significant differences in platelet count and Sokal score according to the transcript type, as shown in Table S1. A total of 113 out of 165 patients (68%) who started IM as frontline treatment obtained sDMR within two years (113 achieved MR^4.0^ and 108 a MR^4.5^), 43 with the e13a2 (38%) and 70 with the e14a2 transcript type (62%). No significant differences were observed between the e13a2 and e14a2 groups regarding the achievement of DMR in patients treated with frontline IM.

**Figure 1:**
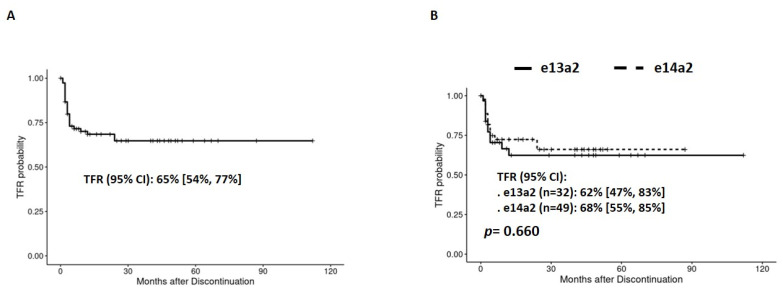
TFR for the present series (**A**) and regarding transcript type (**B**) in IM-treated patients.

Data of TKI discontinuation were available in 81 IM-treated patients (32, 40% with the e13a2 and 49, 60%, with the e14a2 transcript). The probability of TFR (95% confidence interval [CI]) of the whole IM series at 24 months was 65% [54%, 74%] (Figure 1A). Differences in the TFR between the e14a2 and e13a2 transcripts, also at 24 months, were not statistically significant (95% CI: 62% [47%, 83%] vs. 68% [55%, 85%] respectively, *p* = 0.660) (Figure 1B) with an odds ratio (OR) (95% CI) of 1.35 [0.64, 2.93], *p* = 0.439.

### 3.2. IM Treatment Duration and Influence of sDMR on TFR, and by Transcript Type

**Figure 2:**
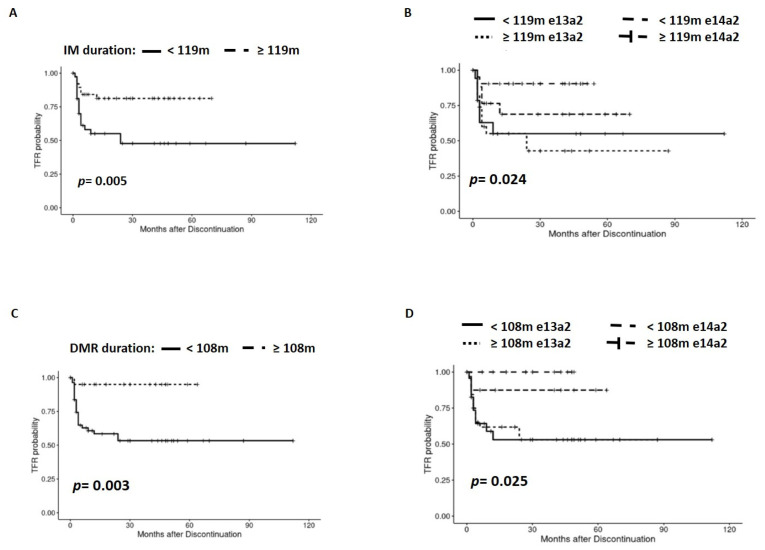
Analysis of the impact of IM treatment duration and sDMR duration, before discontinuation, in TFR for global cohort (**A**,**C**) and regarding transcript type (**B**,**D**).

The duration of IM treatment and sDMR before discontinuation significantly correlated with TFR in our cohort (hazard ratio (HR) [95% CI]: 0.3 [0.13, 0.73], *p* = 0.008 and HR [95% CI]: 0.987 [0.98, 1.00], *p* = 0.046, respectively). Accordingly, we analyzed the cut-off point of the duration of IM treatment and of sDMR that might best discriminate TFR, and we observed that patients treated at least 119 months with IM relapsed less post-discontinuation than those that discontinued IM before completing 119 months (*p* = 0.005) (Figure 2A). In addition, cases with the e14a2 transcript type treated at least 119 months with IM presented a better TFR (*p* = 0.024) (Figure 2B). Similarly, we established a minimum of 108 months of sDMR that also favored TFR (*p* = 0.003) (Figure 2C). This statistical difference was maintained when we separated patients according to the transcript type (*p* = 0.025), being better in patients with the e14a2 transcript type and more than 108 months of sDMR (Figure 2D).

### 3.3. Influence of Age, the Sokal and ELTS Scores on DMR and TFR in IM-Treated Patients

**Figure 3:**
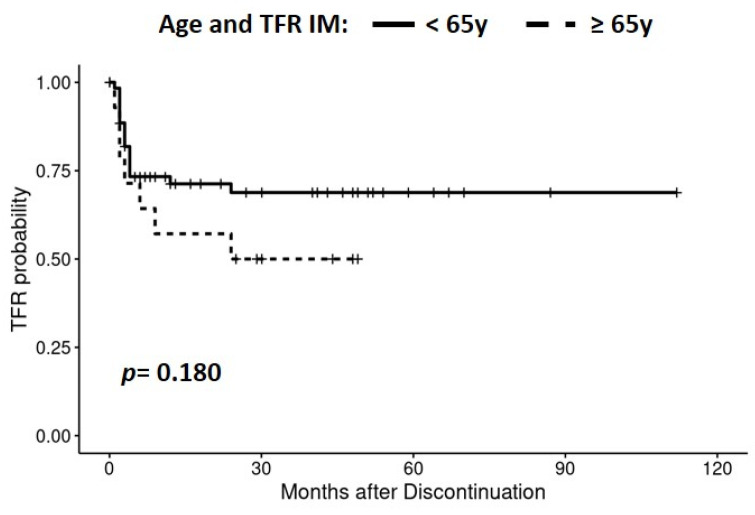
Effect of age on TFR in patients who discontinued IM.

Additionally, we analyzed the influence of age and the Sokal or ELTS scores at diagnosis on TFR in patients that discontinued IM. We did not observe any association between age with a cut-off of 65 years (Figure 3). Similarly, the effect of age on TFR according to the transcript type in IM-treated patients was not relevant. A lack of significance was also observed when analyzing the influence of age on DMR and the Sokal or ELTS score at diagnosis on DMR and TFR, in both the whole series and regarding the two transcript type groups.

### 3.4. Patients Treated with 2G-TKI as Frontline Treatment

We studied 39 CML patients diagnosed from 2008 to 2019, with at least 18 months of follow-up, and who were treated with frontline 2G-TKI treatment (13 DA, 25 NIL and 1 BO) (median: 6 years; 0.3–11.6). At diagnosis, 19 (49%) patients had the e13a2 BCR::ABL1-p210 transcript and 20 (51%) presented the e14a2 transcript. The main demographic and clinical characteristics of the two groups did not significantly differ and neither did the distribution according to ELTS scores. A statistical difference was only observed with blast number and the Sokal score (Table 1).

A total of 37 out of 39 (95%) patients studied at diagnosis presented the t(9; 22) as a single alteration. Other cytogenetic alterations were found in one patient of the e13a2 group and in one of the e14a2 group, but in neither case was there a high-risk alteration. The cumulative incidence of cytogenetic response during the first 3 and 6 months was acquired in 37 out of 39 patients. The complete cytogenetic responses (CCyR) were comparable between the two groups in the two times analyzed. Out of 37 patients studied, 34 and 36 acquired CCyR at 3 and 6 months, respectively. One patient in the e13a2 and two patients in the e14a2 groups did not achieve CCyR at 3 months, while only one patient in the e14a2 group did not reach CCyR at 6 months.

Molecular studies demonstrated that 15 out of 39 patients (38%) (10 with e13a2 and 5 with e14a2) achieved MR^3.0^ at 3 months, while 22 out of 39 (56%) (11 e13a2 and 11 e14a2) did so at 6 months and 34 out of 39 (87%) (17 e13a2 and 17 e14a2) obtained MR^3.0^ at 12 months of 2G-TKI treatment. The transcript type did not influence the acquisition of MR^3.0^ at 3, 6 and 12 months (Table 2).

### 3.5. Effect of Transcript Type on DMR and TFR in 2G-TKI-Treated Patients

**Figure 4:**
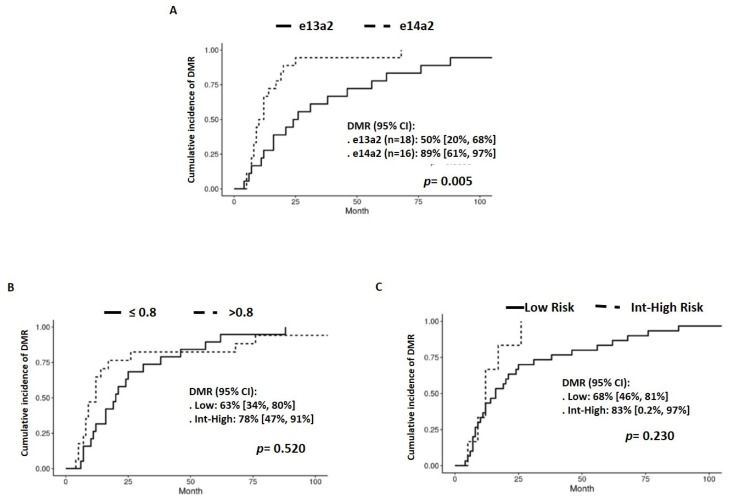
Cumulative incidence of DMR at 2 years in 2G-TKI-treated patients by transcript type (**A**) and considering Sokal and ELTS scores (**B**,**C**) at 2 years, respectively.

A total of 34 out of 39 of the 2G-TKI-treated patients obtained sDMR within two years (32 achieved MR^4.0^ and 33 achieved MR^4.5^). One patient only obtained MR^4^, and two patients achieved MR^4.5^ directly. Patients with the e14a2 transcript type treated with 2G-TKI achieved DMR earlier compared to those with the e13a2 transcript (*p* = 0.005) (Figure 4A). The Sokal and ELTS scores did not influence the achievement of DMR in these two cohorts of patients (*p* = 0.520 and *p* = 0.230, respectively) (Figure 4B,C).

**Figure 5:**
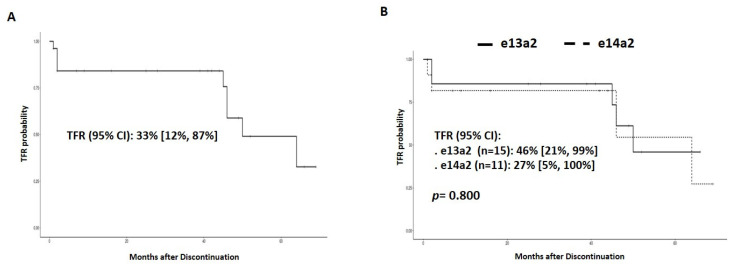
TFR in patients treated with 2G-TKI (**A**) and according to the transcript type (**B**).

Data of TKI discontinuation were available in 26 2G-TKI-treated patients (15 (58%) with the e13a2 transcript and 11 (42%) with e14a2). The probability of TFR at 64 months (95% CI) of the whole 2G-TKI cohort was 33% [95% CI: 12%, 87%]. No significant differences in TFR were observed according to the expression of the e13a2 and e14a2 transcripts (46% [21%, 99%] vs. 27% [5%, 100%], *p* = 0.800) (Figure 5A,B) (OR [95% CI: 0.5, 12.5], *p* = 0.299).

### 3.6. 2G-TKI Treatment Duration and Influence of sDMR on TFR

**Figure 6:**
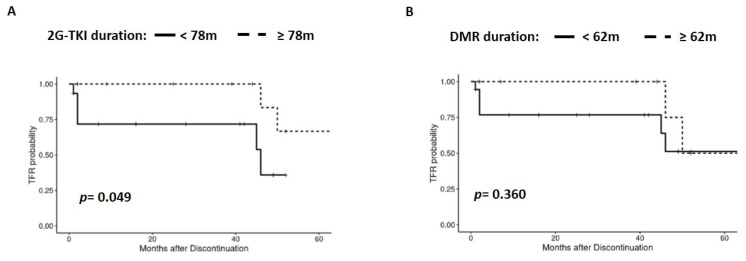
Analysis of TFR according to 2G-TKI treatment duration (**A**) and sDMR (**B**) before stopping treatment.

We observed that patients with at least 78 months of 2G-TKI treatment had a better TFR than those with less than 78 months of treatment (*p* = 0.049) (Figure 6A). Moreover, 62 months of sDMR was the most representative duration affecting TFR (*p* = 0.360) (Figure 6B). The sample size did not allow analysis of these outcomes according to the transcript type.

### 3.7. Influence of Age and the Sokal and ELTS Scores on DMR and TFR in 2G-TKI-Treated Patients

Finally, we also analyzed the influence of age and the Sokal or ELTS scores at diagnosis on TFR in patients that discontinued 2G-TKI. Neither age (>65 y vs. <65 y) nor the Sokal or ELTS scores demonstrated having any influence on TFR in the patients treated with 2G-TKI. Again, the number of patients was insufficient to evaluate TFR according to the expression of the e13a2 and e14a2 transcripts.

## 4. Discussion and Conclusions

Our multicenter analysis of the impact of transcript type on DMR and TFR in CP-CML patients treated with IM or 2G-TKI showed that longer treatment duration and sDMR with IM in patients expressing the e14a2 transcript type positively influenced TFR and that patients with this transcript treated with 2G-TKI achieved DMR earlier than those with the e13a2 transcript.

There are conflicting data as to whether the p210 *BCR::ABL1* transcript type impacts treatment outcomes. Several studies have shown higher rates of cytogenetic and molecular responses in patients with the e14a2 transcript [22,23,24,25,26,27,28,29,30]. Some studies conducted in the Asian population revealed that e14a2 was the most favorable transcript type [19] and had highest response rates in imatinib and nilotinib-treated groups [30]. In the present study, the transcript type did not impact cytogenetic response and MR^3.0^ and sDMR when 2G-TKI were used in frontline treatment, as previously reported [6,19,22]. It is assumed that the higher potency and efficacy of 2G-TKI compared to IM outweigh the possible effect of transcript type on these responses [7,12,13,19,29,31,32]. The difference observed in response rates to nilotinib in our population in respect to the Asian population [30] could be probably explained due to the different number of patients enrolled in the studies rather than ethnicity as there are other European studies with similar results [31,33].

On the other hand, the transcript type of *BCR::ABL1* was not associated with sex, age, splenomegaly, platelet or blast count or the Sokal and ELTS scores in our series of CP-CML patients treated with 2G-TKI, as published by other groups [34,35]. However, we observed a correlation between the e14a2 transcript type and a higher platelet count and the Sokal score in this larger group of IM-treated patients, in line with other groups [21,33,35].

Regarding the influence of transcript type on the acquisition of DMR and sDMR, the data available so far have suggested that patients with the e14a2 transcript have a higher probability of achieving MMR and sDMR [16,17,18,22,30,33], suggesting that e14a2 could be a prognostic factor to guide decisions in CML treatment. In the present analysis, including a larger cohort of patients, we also did not find differences in the percentage of patients treated with IM that acquired DMR and sDMR according to the transcript type, perhaps because some patients were censored when they changed to another TKI, reducing the number of patients evaluable for the analysis. However, it is of note that patients with the e14a2 transcript treated with 2G-TKI achieved DMR earlier than those expressing e13a2, confirming previously reported data. It is hypothesized that the e13a2 transcript confers decreased sensitivity to treatment with TKI, reducing the possibility of achieving or maintaining DMR for more than 2 years. These findings could be explained by the shorter length of the e13a2 protein compared to the e14a2 protein and their differential junctional sequences [16,22,35]. On the other hand, Breccia et al. [17] described an association between Sokal score and sDMR with IM, which was confirmed by our study, as well as with the new ELTS score [8]. In the present study, we did not reproduce the possible impact of the Sokal and ELTS scores on sDMR with the use of IM. When we analyzed the effect of the Sokal and ELTS scores on sDMR in patients receiving 2G-TKI, there was no correlation, which may be explained by the high efficacy of these drugs that offset the influence of these scores on treatment response [7,13,29,31].

The *BCR::ABL1* transcript type may also influence TFR. It has been described that patients with the e14a2 transcript are more likely to maintain TFR than those with e13a2 [16,22,30,36,37]. In the present study, TFR was not influenced by the transcript type in patients treated with IM. Nonetheless, although our data suggest the same conclusion with the use of 2-GTKI, these results must be confirmed in a larger series of patients. However, our results are consistent with what Chen et al. [18] observed in a large cohort of Asian patients treated with TKI, which supports that there are no differences among different regions and ethnic groups. The observed differences may be due to the fact that, according to our hypothesis, the transcript type by itself may not be sufficient to impact TFR, but it may have an effect with a longer duration of TKI treatment or a longer DMR [15,24,38,39,40,41,42]. Pfirrmann et al. [43] reported preliminary data from the EURO-Sky trial and proposed several models and prognostic factors related to MR^3.0^ maintenance at 3 years after stopping TKI. It is of note that the e14a2 transcript was included in one of the models together with the duration of TKI treatment, suggesting that e14a2 has an independent prognostic value in the setting of TFR. Park et al. [42] reported that e14a2 together with longer DMR prior to TKI discontinuation were predictive indicators for successful TFR. In our study, we identified that e14a2 patients treated for more than 119 months with IM and also patients with e14a2 and sDMR longer than 108 months had a better TFR than e13a2 patients. Similarly, patients treated more than 78 months with 2G-TKI were less likely to relapse after TKI discontinuation. These results were consistent with what Chen et al. [3], Park et al. [42] and Mahon et al. [44] described before. They demonstrated that patients with a deeper and longer duration of MR prior to stopping TKI relapsed less. Mahon et al. [44] reported that patients with a median duration of IM therapy of at least 50 months had a higher sDMR rate than those with a shorter median treatment duration, while Park et al. [42] suggested a DMR longer than 48 months for an undetectable e14a2 transcript type by droplet digital PCR and a better TFR.

Some limitations of this study are inherent to its retrospective and multicenter nature. First, the sample size was too small to detect differences between the transcript type and TFR in patients receiving 2G-TKI, and thus, our results should be interpreted with caution. Due to the small number of 2G-TKI-treated patients, we performed the analysis of the three 2G-TKIs together. Second, the time of 2G-TKI treatment in our series could explain the low rate of TFR probability in the 2G-TKI cohort observed in comparison with previous reports in the literature [1,6,44,45] and with IM [46]. Third, it has been suggested that the e13a2 transcript may amplify less due to the presence of a polymorphism in exon 13 of *BCR* that can reduce the efficiency of the primers for the amplification process and this may impact the evaluation of response [26]. Lastly, we cannot rule out the influence of other biological factors on TFR, such as immunological factors, the stem-cell niche and the microenvironment of an individual patient. However, most of the published articles, and ours, strongly support the contention that treatment duration and sDMR duration are two crucial factors that impact TFR [13,15,43,45] and that TFR may be even better in patients with the e14a2 transcript treated longer with TKI, especially IM [16,22,36,37].

In summary, the e14a2 transcript type, together with longer IM treatment and sDMR, favor TFR in CP-CML, and this transcript type is associated with longer and earlier DMR with the use of 2G-TKI.

## Figures and Tables

**Table 1:** Baseline and demographic characteristics of the 2G-TKI patients.

		e13a2 (n = 19)	e14a2 (n = 20)	Total (n = 39)	*p* Value
Sex (male/total) (%)		9/19 (47.4)	11/20 (55.0)	20/39 (51.3)	0.876
Age, median (min, max)		48 (26, 62)	49.5 (25, 78)	48 (25, 78)	0.279
Splenomegaly, median cm (min, max)		1.5 (0, 13)	0 (0, 18)	1 (0, 18)	0.834
Platelets (×10^9^/L), median (min, max)		493 (127, 1080)	463 (338, 1040)	478 (127, 1080)	0.521
PB Blasts (%), median (min, max)		0 (0, 4)	1 (0, 5)	0 (0, 5)	0.071
Cytogenetics, n (%)	t(9;22)	18/19 (94.7)	19/20 (95.0)	37/39 (94.9)	1.000
	t(9;22) + others	1/19 (5.3)	1/20 (5.0)	2/39 (5.1)	
Sokal, n (%)	Low risk	13/19 (68.4)	7/20 (35.0)	20/39 (51.3)	0.077
	Intermediate risk	6/19 (31.6)	12/20 (60.0)	18/39 (46.2)	
	High risk	0/19 (0)	1/20 (5.0)	1/39 (2.6)	
ELTS, n (%)	Low risk	17/19 (89.5)	16/20 (80.0)	33/39 (84.6)	0.342
	Intermediate risk	1/19 (5.3)	59/20 (20.0)	5/20 (12.8)	
	High risk	1/19 (5.3)	0/20 (0)	1/20 (2.6)	

PB: peripheral blood; ELTS: EUTOS long-term survival score.

**Table 2:** Comparison of cytogenetic and molecular response at diferent time points in patients treated with 2G-TKI.

		e13a2	e14a2	Total	*p* Value
CCyR	3 months (n)	49% (18)	43% (16)	92% (34)	0.603
	6 months (n)	51% (19)	46% (17)	97% (36)	0.487
MR^3.0^	3 months (n)	33% (10)	17% (5)	50% (15)	0.462
	6 months (n)	41% (11)	41% (11)	82% (22)	0.341
	12 months (n)	46% (17)	46% (17)	92% (34)	1.000

CCyR: complete cytogenetic response; MR^3.0^: major molecular response.

## Data Availability

The data present in the study can be partially available upon the request to corresponding author, under national regulations for data sharing.

## Supplementary Materials

The following supporting information can be downloaded at: https://www.mdpi.com/article/10.3390/jcm13030779/s1, Table S1: Demographic and baseline clinical characteristics of the IM study series.
