# Supplementary material for: e14a2 Transcript Favors Treatment-Free Remission in Chronic Myeloid Leukemia When Associated with Longer Treatment with Tyrosine Kinase Inhibitors and Sustained Deep Molecular Response
Source: J Clin Med. 2024 Jan 29;13(3):779. doi: 10.3390/jcm13030779 (PMC10856594; doi:10.3390/jcm13030779)
Supplement: Supplementary file 1 [file jcm-13-00779-s001.zip › jcm-2797965-supplementary.pdf]

Table S1. Demographic and baseline clinical characteristics of the IM study series.

		e13a2 (n=86)	e14a2 (n=138)	Total (n=224)	*p* value
**Sex (male/total) (%)**		42/86 (48.8)	71/138 (51.4)	113/224 (50.4)	0.808
**Age, median (min, max)**		54 (15, 83)	56 (12, 84)	55 (12, 84)	0.164
**SplenomegalyP, median cm (min, max)**		0 (0, 20)	0 (0, 30)	0 (0, 30)	0.260
**Platelets (x10e9/L), median (min, max)**		312 (37, 2240)	382 (1, 1870)	362 (1, 2240)	0.016
**Blasts PB (%), median (min, max)**		0 (0, 11.0)	0 (0, 11.0)	0 (0, 11.0)	0.548
**Cytogenetics, n (%)**	**t(9;22) (%)**	67/74 (90.5)	110/127 (86.6)	177/224 (88.1)	0.547
	**t(9;22) + others (%)**	7/74 (9.5)	17/127 (13.4)	24/224 (11.9)	
**Sokal, n (%)**	**Low risk (%)**	48/85 (56.5)	50/132 (37.9)	98/224 (45.2)	0.027
	**Int risk (%)**	28/85 (32.9)	61/132 (46.2)	89/224 (41.0)	
	**High risk (%)**	9/85(10.6)	21/132 (15.9)	30/224 (13.8)	
**ELTS, n (%)**	**Low risk (%)**	53/85(62.4)	70/132 (53.0)	123/224 (56.7)	0.267
	**Int risk (%)**	26/85 (30.6)	45/132 (34.1)	71/224 (32.7)	
	**High risk (%)**	6/85 (7.1)	17/132 (12.9)	23/224 (10.6)	
